# Focal segmental glomerulosclerosis associated with mitochondrial disease 

**DOI:** 10.5414/CNCS109083

**Published:** 2017-03-03

**Authors:** Kenneth Lim, David Steele, Andrew Fenves, Ravi Thadhani, Eliot Heher, Amel Karaa

**Affiliations:** 1 Division of Nephrology, and; 2 Department of Genetics and Pediatrics, Massachusetts General Hospital, Harvard Medical School, Boston, MA, USA

**Keywords:** focal segmental glomerulosclerosis (FSGS), kidney transplantation, mitochondrial disease, hearing loss, deafness, whole exome sequencing

## Abstract

Primary mitochondrial diseases (MD) are complex, heterogeneous inherited diseases caused by mutations in either the mitochondrial or nuclear DNA. Glomerular diseases in MD have been reported with tRNA mutation m.3243A>G causing a syndrome of mitochondrial encephalomyopathy, lactic acidosis and stroke-like episodes (MELAS). We describe here a case of focal segmental glomerulosclerosis (FSGS) associated with a new tRNA mutation site. A 34-year-old man with a history of living related kidney transplantation, diabetes, hearing loss, and developmental delay presented to the outpatient clinic with complaints of new behavioral difficulties, worsening symptoms, and brain involvement on imaging. Physical examination was remarkable for difficulty hearing, a pattern of dysarthric speech, and cerebellar gait. Laboratory investigations including lactate levels were unremarkable. Based on this set of clinical circumstances, concern for an underlying genetic abnormality was raised. Multiple metabolic tests were unremarkable. Whole exome sequencing revealed a mitochondrial MT-TW tRNA change at position m.5538G>A. Genotype-phenotype correlations are consistent with this tRNA mutation as a cause of his symptoms. To the best of our knowledge, this is the first case describing FSGS-associated MD caused by an m.5538 G>A mutation. Consideration of an underlying MD should be made in patients presenting with deafness, neurologic changes, diabetes, and renal failure.

## Introduction 

Friedrich Nietzsche (1844 – 1900) was perhaps one of the most influential German philosophers in history [1]. His medical biography has intrigued many for decades, exemplifying a constellation of seemingly unrelated symptoms involving multiple organ systems, and has been a subject of controversy. Today, his symptoms have been widely thought to be secondary to a form of mitochondrial disease (MD) called MELAS syndrome that includes mitochondrial encephalopathy, lactic acidosis, and stroke-like episodes. MELAS syndrome is associated in ~ 80% of cases with a mitochondrial gene mutation at position m.3243A>G [2]. Primary MDs are complex, heterogeneous inherited diseases and they can be caused by mutations in either the mitochondrial or nuclear DNA. Mitochondria are transferred from mothers to their progeny in the oocyte and therefore genetic conditions involving the mitochondrial DNA follow a maternal inheritance [3]. Phenotypic expression of MDs can be highly variable [3] in part reflecting the ubiquitous distribution of mitochondria potentially affecting all tissues and organs. 

Over the past two decades, several patterns of renal diseases have been associated with MDs, including glomerular disease, tubulopathies, cystic disease, and tubulointerstitial nephritis [4]. The majority of patients diagnosed with MD-associated renal disease are in their second or third decade of life and over half of these patients have chronic kidney disease (CKD) [3]. While renal involvement in MELAS syndrome is uncommon, patients with m.3243A>G mutations can develop renal failure and proteinuria usually in association with diabetes, sensorineural hearing loss, and other neuromuscular deficits. It is estimated that ~ 1% of the diabetic population in Europe and Japan carry this genetic defect [5]. Approximately 80% of patients with renal involvement have a hearing impairment and these patients can be misleadingly diagnosed with Alport’s syndrome [6]. The prevalent histological renal pathology finding in these patients with mitochondrial DNA point mutations is focal segmental glomerulosclerosis (FSGS) [7]. 

Only a rare few other mitochondrial point mutations have been described to be associated with FSGS, and these are located at m.4269A>G, m.5728A>G, and m.A5843A>G [8]. Patients with mitochondrial DNA deletions that cause Kearns-Sayre [9, 10] and Pearson syndromes [11] rarely have glomerular pathologies and most often develop renal tubular dysfunction and interstitial nephritis. We describe here a case of FSGS-associated MD caused by an m.5538G>A mutation, which to the best of our knowledge is the first of its kind. An additional unique feature of this is the complex multiorgan involvement on a background of developmental delay, hearing loss, diabetes, and renal failure. 

## Case report 

A 34-year-old man with a history of end-stage renal disease (ESRD) who underwent living related kidney transplantation in 2005 and had diabetes, hearing loss, hypertension, and mild developmental delay presented to the outpatient clinic with complaints of static encephalopathy (permanent or unchanging brain dysfunction) and worsening MRI findings of progressive cortical and subcortical atrophy, calcification of the bilateral basal ganglia and cerebellar dentate nuclei, subcortical white matter changes, and corpus callosum lesions. At age 12, he first noted bilateral progressive hearing decline secondary to a sensorineural deficit. At age 14, he developed non-nephrotic range proteinuria and underwent kidney biopsy that showed FSGS. He subsequently developed ESRD and required hemodialysis prior to receiving a living related kidney transplant from his father by age 24. Following his kidney transplantation, he developed steroid-induced diabetes. His kidney transplant course has otherwise been uncomplicated and he has had no indication for an allograft biopsy. At the time of his clinic visit with us, his mother noted him to have new behavioral difficulties, specifically disorganized thought processes with complaints of disorderly conduct likely related to worsening brain abnormalities and raising concern for an underlying genetic abnormality. During evaluation, the patient also suffered a stroke of the posterior right lentiform nucleus and posterior limb of the right internal capsule. 

**Figure 1.**
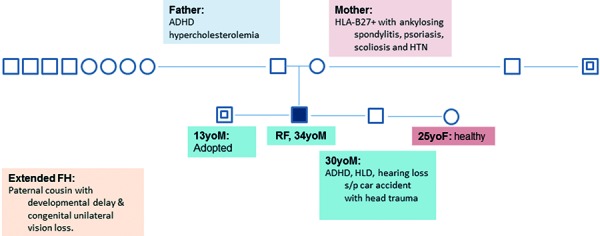
Family pedigree.

His social history was negative for tobacco, alcohol, or illicit drug usage. The patient was able to finish high school and attend a year in community college. He was the product of a normal pregnancy after a prior miscarriage. His family pedigree is illustrated in Figure 1. His medications included mycophenolate mofetil, tacrolimus, gemfibrozil, esomeprazole, and vitamin D. He has no allergies. Physical examination was remarkable for a mild cerebellar gait, decreased hearing, otherwise all other systems were largely negative including normal retinal exam. 

**Figure 2.**
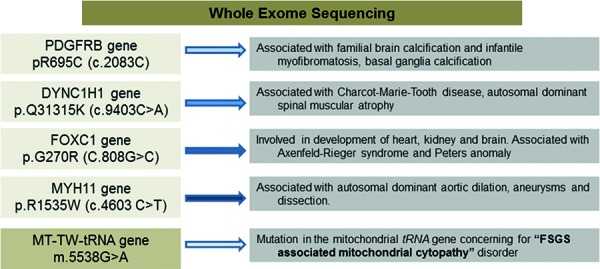
Results of whole exome sequencing.

Laboratory investigations including lactate levels and other metabolic profiles were unremarkable. Whole exome sequencing revealed a mitochondrial MT-TW tRNA change at position m.5538G>A with 30% heteroplasmy (HTP) in blood as well as several variants of unknown significance in genes associated to diseases that do not correlate with patient’s phenotype or may only have a slight contribution to symptoms as illustrated in Figure 2. Genotype-phenotype correlation in this case is most consistent with this tRNA mutation as a cause of this patient’s symptoms. 

## Discussion 

There is a paucity of data on genetic mitochondrial defects and their renal manifestations both in pediatric patients, and even more so in adults [4]. This is reflective of their designation as rare diseases, phenotypic, and genotypic heterogeneity making them difficult to recognize and begging the need for initiatives to raise awareness, and improve diagnostic and therapeutic strategies for the care of patients afflicted with MDs. Organ systems and tissue types that are most severely affected by MDs are those that are reliant on aerobic metabolism for ATP production and oxidative phosphorylation, and this is particularly the case for the central nervous system and skeletal muscle. Renal manifestations in MDs are rarely described in isolation and therefore the coexistence of neuromuscular symptoms and renal manifestations should raise the suspicion of an underlying MD [3]. Identifying specific mutation sites involved in mitochondrial-associated renal diseases can help inform our diagnostic approaches while yielding valuable insight into the genetics of these conditions. The case presented here therefore provides critical information on a new mitochondrial MT-TW tRNA change at position m.5538G>A that is potentially associated with the development of FSGS. 

The m.3243A>G point mutation is the most commonly described mutation to date in MDs that has been linked to renal disease. In our case, whole exome sequencing did not reveal the presence of this point mutation. Several other gene variants were revealed in our patient (Figure 2), however following literature review, genetic database consultation and detailed phenotyping of patient, the known conditions associated with these variants were not present in our case. Variant in the PDGFRB gene found in the case is common in the general population and is associated with familial brain calcification, however this variant could not account for the constellation of the other phenotypic changes that were present in our patient. Additionally, none of these other variants have been associated with FSGS to the best of our knowledge. 

Mitochondrial tRNA mutations have been involved in a host of syndromes ranging from single symptom to multi-systemic disease. The tRNA^Trp^ mutation found in this patient has been linked to mitochondrial myopathy, Leigh syndrome, myoclonic epilepsy, and various encephalomyopathies [12]. Skeletal muscles usually reveal ragged red fibers, cytochrome C oxidase negative fibers, abnormal mitochondria on electron microscopy, and electron transport chain deficiencies on biochemical testing [13]. Due to our patient’s frail condition and multiple co-morbidities, a muscle biopsy was declined. Mitochondrial function tests such as respiratory chain enzyme activities can be performed on fresh renal tissue, but this is mostly available on a research basis [4]. Tissue can also be obtained from other sources such as in blood peripheral leukocytes and skin fibroblasts for functional analysis however, MDs and their manifestations can be tissue specific and therefore some cases can be missed. A limitation of our case presented is that we were unable to obtain kidney tissue for functional analysis. Serum lactate levels have generally been measured in cases of suspected MDs, however lactate levels can be normal such as in our patient [4]. This means that the diagnosis of MDs relies upon recognition of clinical manifestations and information obtained from a detailed family history ultimately confirmed by molecular testing. Due to these factors, the incidence of renal-associated MDs is in all likelihood underestimated. Known mitochondrial tRNA mutations that have been associated with kidney disease are summarized in Table 1. 

Interestingly, 2 other patients who have been reported carrying the m.5538G>A mutation had very different symptoms compared to our patient which included myoclonic epilepsy, cataract, pigmentary retinopathy, hypothyroidism, and mild myopathy in the proband (65% HTP in muscle, 5% in blood) and only hypothyroidism and diabetes mellitus in the proband’s mother (20% HTP in muscle) [13]. This discrepancy is not uncommon with mtDNA disease and may be related to mutation heteroplasmy level and tissue segregation. Our patient was noted to develop diabetes after beginning steroid therapy as part of his immunosuppressive regimen. However, it is likely that his mitochondrial disease predisposed him to develop diabetes at such an early age. We also note that other anti-codon stem mutations in the mitochondrial transfer RNA^Trp^ at position m.5540G>A present with early onset hearing loss, abnormal brain imaging including basal ganglia calcification, and marked overall brain atrophy with white matter changes very much similar to our case [14]. None of the patients reported with the m.5540G>A mutation had any renal involvement but they were young in age compared to our patient and kidney involvement may have been missed at the time of the initial evaluation. 

Due to the association of MDs with deafness and difficulties with defining clear inheritance patterns in some cases, patients with MDs can be mistakenly diagnosed with Alport’s syndrome [5]. On review of two studies that screened a total of 90 patients with Alport’s syndrome for the MELAS mutation, 2 patients were found to be misdiagnosed [5, 15]. In MDs, hematuria is usually absent and disruption of the glomerular basement-membrane characteristic of Alport’s syndrome on renal histopathology is also absent. Therefore, the presence or absence of hematuria may help to distinguish between MDs and Alport’s syndrome. Of note, patients with Alport’s syndrome can present with FSGS on kidney biopsy and therefore, this cannot be used as a feature to distinguish between the two conditions [16, 17]. Mutations in the *COL4A3-5* genes that encode α-chains of glomerular basement membrane collage type IV are classically associated with Alport’s syndrome, and recent studies have shown that these mutations can be associated with FSGS [18]. In our patient, whole exome sequencing did not reveal *COL4A3-5* mutations. 

While no cure currently exists for the management of MD-associated renal disease, organ transplantation remains a therapeutic option as in the case of our patient. Patients with MD are generally at risk for worsening of their disease by the catabolic stress associated with transplantation and associated risks of infection or medication-related toxicity. However, in a recent case series that examined the largest group of post-transplant patients (35 patients) with primary MDs to-date who underwent either liver, kidney, or heart transplantation, the majority of patients did not have worsening of their MD within 90 days of their transplantation [19]. The authors also reported that post-transplant complications, including organ rejection, were not a common occurrence. Our patient has had an uncomplicated post-transplant course following a living related kidney transplant from his father. As mitochondrial DNA diseases are maternally transmitted, we would not expect the father to carry the same DNA change. If the diagnosis of maternally inherited mitochondrial disease is known prior to transplant, maternal family donors should be avoided as they may also carry the mutation. 

In conclusion, consideration of an underlying MD should be made in patients presenting with the constellation of deafness, neurologic changes, diabetes, and proteinuric renal failure. The case presented here adds to our current understanding of MD-associated renal disease and is the first to provide genotype-phenotype evidence of a new FSGS-associated MD located at position m.5538 G>A. 

## Funding sources 

None. 

## Conflict of interest 

All authors declare no conflicts of interests. 

**Table 1.** Mitochondrial DNA mutations associated with kidney disease.

Mutation	Gene	Renal phenotype	Reference
m.G5538A	mt-tRNA	FSGS	First described in this case report
m.G586A	mt-tRNA	TIN	[20]
m.A608G	mt-tRNA	TIN	[21]
m.G3242A	mt-tRNA	Renal failure, RTA type 4	[22]
m.A3243G	mt-tRNA	FSGS	[2, 8]
m.A3243G	mt-tRNA	TIN	[23, 24]
m.A4269G	mt-tRNA	FSGS	[25]
m.A5656G	Noncoding region	TIN	[26]
m.A5728G	mt-tRNA	FSGS	[27]
m.A5843G	mt-tRNA	FSGS	[28]
m.12425delA	NADH dehydrogenase 5	Glomerulocystic disease, renal failure	[29]

Mt-tRNA = mitochondrial transfer RNA; FSGS = focal segmental glomerulosclerosis; TIN = tubulointerstitial nephritis.
